# Evaluating the Efficacy of a Mobile App (Drinks:Ration) and Personalized Text and Push Messaging to Reduce Alcohol Consumption in a Veteran Population: Protocol for a Randomized Controlled Trial

**DOI:** 10.2196/19720

**Published:** 2020-10-02

**Authors:** Daniel Leightley, Roberto J Rona, James Shearer, Charlotte Williamson, Cerisse Gunasinghe, Amos Simms, Nicola T Fear, Laura Goodwin, Dominic Murphy

**Affiliations:** 1 King's College London King's Centre for Military Health Research London United Kingdom; 2 King's College London King’s Health Economics London United Kingdom; 3 Combat Stress Leatherhead United Kingdom; 4 King's College London Department of Psychological Medicine London United Kingdom; 5 Academic Department of Military Mental Health King's College London London United Kingdom; 6 British Army London United Kingdom; 7 University of Liverpool Department of Psychological Sciences Liverpool United Kingdom

**Keywords:** alcohol misuse, smartphone, intervention, SMS text messaging, push notifications

## Abstract

**Background:**

Alcohol misuse is higher in the UK Armed Forces than in the general population. Previous research has shown that interventions delivered via smartphones are efficacious in promoting self-monitoring of alcohol use, have utility in reducing alcohol consumption, and have a broad reach.

**Objective:**

This single-blinded randomized controlled trial (RCT) aims to assess the efficacy of a 28-day brief alcohol intervention delivered via a smartphone app (*Drinks*:Ration) in reducing weekly self-reported alcohol consumption between baseline and 3-month follow-up among veterans who drink at a hazardous or harmful level and receive or have received support for mental health symptoms in a clinical setting.

**Methods:**

In this two-arm, single-blinded RCT, a smartphone app that includes interactive features designed to enhance participants’ motivation and personalized messaging is compared with a smartphone app that provides only government guidance on alcohol consumption. The trial will be conducted in a veteran population that has sought help through Combat Stress, a UK veteran’s mental health charity. Recruitment, consent, and data collection will be carried out automatically through the *Drinks*:Ration platform. The primary outcome is the change in self-reported weekly alcohol consumption between baseline (day 0) and 3-month follow-up (day 84) as measured using the Time-Line Follow back for Alcohol Consumption. Secondary outcome measures include (1) change in the baseline to 3-month follow-up (day 84) Alcohol Use Disorder Identification Test score and (2) change in the baseline to 3-month follow-up (day 84) World Health Organization Quality of Life-BREF score to assess the quality of adjusted life years. Process evaluation measures include (1) app use and (2) usability ratings as measured by the mHealth App Usability Questionnaire. The primary and secondary outcomes will also be reassessed at the 6-month follow-up (day 168) to assess the longer-term benefits of the intervention, which will be reported as a secondary outcome.

**Results:**

The study will begin recruitment in October 2020 and is expected to require 12 months to complete. The study results will be published in 2022.

**Conclusions:**

This study assesses whether a smartphone app is efficacious in reducing self-reported alcohol consumption in a veteran population that has sought help through Combat Stress using personalized messaging and interactive features. This innovative approach, if successful, may provide a means to deliver a low-cost health promotion program that has the potential to reach large groups, in particular those who are geographically dispersed, such as military personnel.

**Trial Registration:**

ClinicalTrials.gov NCT04494594; https://clinicaltrials.gov/ct2/show/NCT04494594

**International Registered Report Identifier (IRRID):**

PRR1-10.2196/19720

## Introduction

### Alcohol Misuse Burden

Alcohol misuse is common in the UK Armed Forces (AF), with the prevalence higher in the AF than in the general population [1-4]. Research indicates that this trend continues after personnel leave service (*veteran* or *ex-serving* is used interchangeably in the UK) [1,5]. It has been estimated that more than 50% of those who have left the AF meet the criteria for hazardous alcohol use, a score of 8 or above on the Alcohol Use Disorders Identification Test (AUDIT) [3,6].

Research has shown that individuals in the general population underestimate their drinking and do not perceive it as problematic, even at potentially harmful levels [7]. Young males are at particular risk of underestimating their drinking [7]. This pattern is similar among the UK AF, with less than half of UK AF hazardous drinkers recognizing that they have an alcohol problem [8]. There is also a culture of heavy alcohol use in the UK AF, which is encouraged by the social environment and carries on after personnel leave service [4,9,10]. Therefore, leaving service could provide an opportunity to initiate behavior change in settings with less peer pressure to conform to social norms and promote alcohol awareness.

Focusing on personnel who have left service, a similar pattern has emerged for those who are seeking support for mental health problems and consume alcohol to cope. A recent study of treatment-seeking UK service leavers identified that 43% of respondents reported misusing alcohol and that alcohol misuse was commonly comorbid with posttraumatic stress disorder (PTSD) and common mental health difficulties such as anxiety and depression [11]. Alcohol misuse can also have an impact on treatment adherence. Treatment-seeking veterans with alcohol misuse attended fewer mental health appointments and were more likely to have a negative perception of mental health treatment when alcohol misuse is comorbid with depression or PTSD [12]. It is, therefore, important to develop interventions that may target drinking to cope with motivations, which may subsequently enhance engagement with mental health services and improve mental health outcomes.

The impact of alcohol misuse by AF personnel on the wider society (eg, health care utilization, productivity, and welfare) is unknown; however, it is likely to be increased compared with the general population. Research has indicated that heavy drinking in England, which is frequently comorbid with mental health difficulties [13], is estimated to cost the National Health Service (NHS) of the UK £3.5 billion (US $4.4 billion) per year (3.6% of its annual budget) [14].

### Brief Interventions

There is a range of effective intervention options for management and treatment of alcohol misuse categorized as brief interventions, specialist treatment, and less intensive treatments that span the two [15]. Personnel of the UK AF who have left service typically access the same health care system as the general public, and the same interventions are offered to both groups.

Brief interventions for alcohol misuse are a popular management and treatment option used in the UK general population, often provided to individuals scoring 15 or below on the AUDIT [16]. A common theme among these interventions is the goal of improving recognition, targeting the individual’s motivations to reduce their alcohol consumption, and developing coping strategies to control and reduce intake [15-17]. This includes motivational interviewing, cognitive behavioral therapy, behavior self-control training, behavior change, and coping development, all of which are often tailored to the individual by a clinician [15].

Brief interventions aim to raise awareness of the risks associated with alcohol misuse and help individuals reduce their hazardous drinking. Often brief interventions are delivered to individuals who are not seeking help for alcohol misuse from a specialist alcohol service and are delivered in the general community setting (eg, general practitioners, hospital doctors, and nurses). There is a body of research that confirms the efficacy of brief interventions in reducing alcohol consumption and alcohol-related harm for those drinking at a hazardous level in the general population [18-20]. However, little is known about their efficacy in the AF context [19]. Given the shared culture in the military in which alcohol plays a meaningful role, it is important to evaluate the efficacy of brief interventions in this type of population.

### Smartphone-Based Brief Interventions for Alcohol Misuse

The last two decades have seen a proliferation in the use of digital technologies to support brief intervention management and treatment of alcohol misuse in the general public; however, little attention has been paid to the AF community [21-24]. In the late 1990s, interventions were commonly delivered via a computer using CD-ROM–based programs. However, with the advent of the World Wide Web, many new opportunities arose to harness increased reach, provide real-time monitoring, and offer personalized treatment [25-31]. This includes the use of SMS, which has been shown to be effective in encouraging people to change their behavior [32-34].

Over the last 5 years, the mode of intervention delivery has shifted from web- to smartphone-based [35]. Smartphone interventions for alcohol misuse, such as *Drink Less* [24] and *Drink Aware* [36], have several advantages over web-based interventions. These include having a low cost per use, allowing for rapid changes and iterative development, avoiding the stigma associated with receiving help in person, and are highly convenient because they can be used as and when the individual wants (discreetly or openly). However, these smartphone interventions are focused on the general population and do not target individual beliefs, prevailing social contexts, and perceptions of consumption experienced by the AF community [9,37]. Smartphone-based interventions allow users to revisit information about their drinking habits as often as they need to and, thus, have the potential to promote positive behavioral changes [18,38]. Furthermore, there are indications of the potential of smartphone-based interventions being cost effective if found to be efficacious [25,28,29].

Most existing alcohol apps targeted at the general public include self-monitoring (eg, *Drink Less* [24], *Drink Aware* [36], and One You Drinks Tracker [39]), whereby users are encouraged to regularly record and monitor (via visual graphics) their alcohol consumption within an app [24,40]. Self-monitoring (or self-recording) has been found to be associated with improved outcomes and an effective behavior change technique (BCT) for reducing alcohol use. A BCT is defined as a specific component of an intervention designed to change behavior and a putative active ingredient in an intervention [21]. A review of personalized digital interventions for reducing hazardous and harmful alcohol consumption found that providing behavior substitution, problem solving, and providing a credible source were associated with better outcomes, including reductions in alcohol consumption [41]. There are benefits to the use of digital technology for the UK AF community. However, to date, there is no published work that seeks to test a brief intervention alcohol reduction app that is personalized to individual users or targeted to support military veterans.

### Study Aim

The aim of this randomized controlled trial (RCT) is to assess the efficacy of a 28-day brief alcohol intervention delivered via a smartphone app (*Drinks*:Ration) in reducing self-reported weekly alcohol consumption between baseline and 3-month follow-up (day 84) among veterans who drink at a hazardous or harmful level and receive or have received support for mental health symptoms in a clinical setting.

## Methods

### Ethical Approval

This study was approved by the local ethics committee of King’s College London (registration number: HR-19/20-17438) and registered as a clinical trial (registration number: NCT04494594).

### Study Design

**Figure 1:**
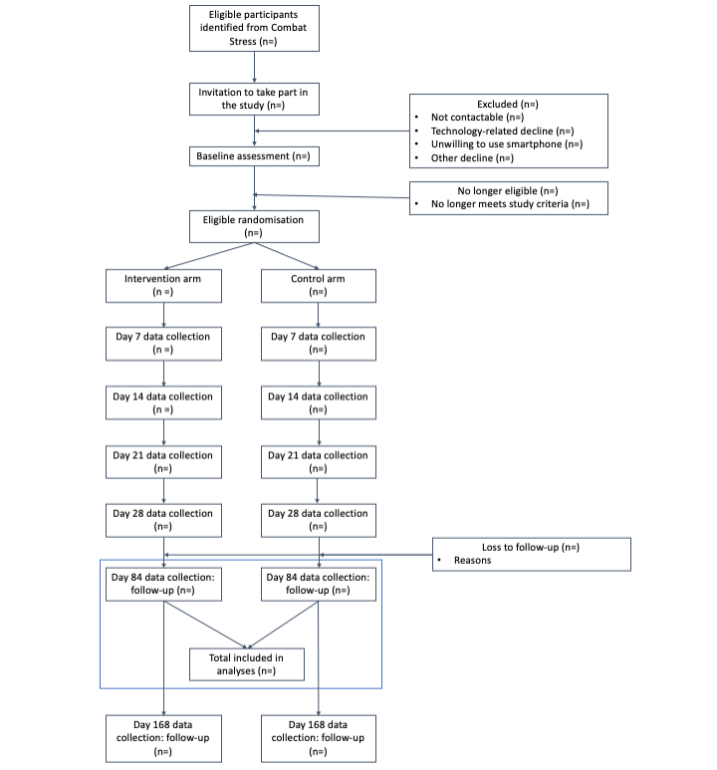
Study flow diagram. Data collected on day 7/14/21 are required to tailor and personalize the messaging of the Drinks:Ration app for the intervention arm. Primary and secondary outcomes are assessed at day 84, with the outcomes reassessed at day 168 to assess the longer-term benefits of the intervention.

This is a two-arm participant-blinded (single-blinded) RCT, which compares a smartphone app (control arm) that includes feedback on baseline self-reported alcohol consumption with a smartphone app (intervention arm) that includes individualized normative feedback, in addition to features designed to enhance participants’ motivation, app interactive feedback, and self-efficacy in modifying their alcohol consumption. We hypothesize that the intervention arm app will be efficacious in reducing alcohol consumption compared with the control arm app. In this study, both the control and intervention arms will be delivered via one smartphone app known as *Drinks*:Ration. Participants in the control arm are given access only to the alcohol consumption feedback on the basis of publicly available health guidance [42] and reminder messaging to consult the feedback. Those in the intervention arm are given full access to the app, which includes all theoretically driven components and messaging (discussed further in the Intervention section). Both arms will be asked to use the app for 28 days. This study is designed such that the control arm structurally resembles the intervention arm but lacks the active ingredients (eg, drinks diary, interactive drinking feedback, and goal setting). This approach was selected to ensure (1) that both groups receive a digital intervention, (2) uniformity and provide relevance to real-world interventions, and (3) that participants are blinded from the study arm they are assigned to. The trial is conducted via the *Drinks*:Ration platform without any need for involvement of the research team, including the collection of informed consent and data collection. The study flow and data collection points are shown in Figure 1.

### Study Sample

Participants may have been, or are presently, receiving treatment through Combat Stress. Treatment offered via Combat Stress is for depression, anxiety, and PTSD. However, Combat Stress does not offer alcohol treatment services. The eligibility criteria are as follows:

#### Inclusion Criteria

Participants will be included in the study if they have downloaded the app onto an iOS or an Android device, are aged 18 years or older, live in the United Kingdom, consume 14 UK units (approximately 140 g) of alcohol or more per week as measured using Timeline Follow-back for alcohol consumption (TLFB) [43] at baseline (day 0), provide a mobile phone number, and are veterans of the UK AF. A minimum threshold of 14 UK units of alcohol per week is used in this study to reflect scientific research and UK national guidelines that indicate that regular drinking of more than this threshold risks damage to health [44,45]. It is important to note that in the United Kingdom, individuals are defined as veterans if they have completed a minimum of 1 day paid employment in the UK AF.

#### Exclusion Criteria

Participants will be excluded if they are listed as being *red* risk by Combat Stress, which is determined by the clinical team following an initial assessment and is based on a traffic light system to assess risk, where red indicates an immediate high risk to the individual and/or others. *Red* risk is applied independently of the research team and is used only where participation in the study could impact clinical treatment. The exclusion applies only during the contact list data extraction. Participants will be excluded if they do not have a mobile phone or have not given the Combat Stress consent for contact for research purposes.

A power calculation was performed based on previously reported data from Combat Stress [11,46]. This study requires a sample of 37 participants in each arm, with an 80% power to detect with a probability of 5% mean change in alcohol consumption of 4 UK units (approximately 40 g of alcohol per week, 10 g per unit) between control and intervention arms, at 3-month follow-up (day 84). Four units have been selected based on these 2 criteria: (1) reductions observed in similar studies [4,8,22,24,47] and (2) reductions observed in the feasibility trial of *Drinks*:Ration, which found a 7 UK-unit decrease at week 4. We aim to invite a minimum of 620 veterans to participate in the study to account for a 30% acceptance of invitation and 40% attrition rate.

### Recruitment

This study will recruit participants through Combat Stress (established in 1919), a third-sector charitable organization that provides mental health services, including substance misuse to UK veterans. It is the largest military charity in the United Kingdom in terms of the number of individuals treated, providing both inpatient and outpatient secondary mental health services to veterans and specializing in PTSD. Since 2011, Combat Stress has been funded by the NHS in the United Kingdom to provide a national specialist PTSD clinical service for veterans [48]. Combat Stress facilitates access to eligible participants and jointly with King’s College London oversees any risks that arise as a result of participation in the study. It is important to note that although this study uses a sample of veterans from Combat Stress, it is hoped that *Drinks*:Ration will be useful for the wider veteran community.

Potential participant contact details (postal address and/or email if available) will be extracted from the Combat Stress Patient Management System for those who meet the study eligibility criteria and who have provided consent to be contacted for research purposes. Only those who have had contact with Combat Stress over a 2-year period between January 1, 2018, and December 31, 2019, will be contacted. It is estimated that about 3000 patients with Combat Stress are eligible to be contacted by the research team.

Where contact details are available, eligible participants will be invited to take part via email in the first instance. The first contact will include an explanation of the study, link to the participant information sheet, and instructions on how to download *Drinks*:Ration using a unique quick response (QR) code. Once participants have downloaded the app, they will be invited to report alcohol consumption using TLFB for alcohol consumption [43] for the last 7 days and confirm the military serving status (to validate eligibility). Those meeting the study eligibility criteria will be invited to register an account and complete the baseline questionnaire (day 0).

### Randomization and Blinding

Randomization occurs when a QR code is generated and a unique proxy identifier and participant gender are assigned. At this point, participants are automatically randomized to receive the control or intervention arm (see the Intervention section) and are blinded. Block randomization of size 2 will be used to ensure equal gender distribution across both conditions. The randomization procedure is carried out automatically by the *Drinks*:Ration platform with no human involvement, except to provide a proxy identifier and gender. The research team will be blinded to the randomization of the participant, except for DL, who led the development and will manage the *Drinks*:Ration app throughout the study.

### Intervention

The *Drinks*:Ration app, formerly called *InDEx* [49-51], has been developed following the Medical Research Council Complex Intervention Guidelines and using co-design methodology. It has been developed by the King’s Centre for Military Health Research (at King’s College London) and the University of Liverpool, supported by experts in smartphone app development, epidemiology, addiction psychiatry, and military mental health. The app is designed to support veterans drinking at a hazardous or harmful level by providing bespoke advice and support over a 28-day period. The app is designed to enhance participants’ motivation and self-efficacy in modifying their alcohol consumption by means of BCT in the content displayed (Multimedia Appendix 1) and the messaging sent to participants. The iterative development process, theoretical framework, and feasibility trial have already been reported [49-51]. Briefly, *Drinks*:Ration has been developed and tested with the following 5 core modules:

1. Account management: participants can modify personal information (eg, first name and mobile number) and app parameters (eg, automatic logout, clear local storage, data-sharing permission, and exiting the study).
2. Questionnaire and individualized normative feedback: it captures the participant’s responses to a set of questions and aggregates responses to produce an individualized infographic that represent the participant’s alcohol consumption compared with the general population, the AF Community, and other participants of *Drinks*:Ration.
3. Self-monitoring and feedback: it records alcohol consumption by participants and provides a range of visual illustrations (eg, charts, figures, and texts) to allow for monitoring of alcohol consumption. Moreover, the participants can select visual metrics relevant to their interest (eg, calories, cost, and exercise required).
4. Goals (setting and review): participants can set goal(s) based on the implementation intentions (if and then) [52] methodology; visual feedback provides feedback on progress toward achieving set goal(s).
5. Personalized messaging: participants are sent tailored messages via push notification or SMS text messaging that provides prompts to use the drinks diary, suggests alternative behaviors, and provides feedback on set goals.

The app is compatible with all modern iOS- and Android-supported devices, and no involvement of the research team is required for participant enrolment. Data is collected, managed, and processed using the Google Firebase server infrastructure in London, United Kingdom.

In this study, the intervention arm will receive the app with all functionalities, including push notifications and SMS text messaging. The control arm participants will have access to only the *questionnaire and feedback* module and will not receive any form of personalized messaging, except prompts to complete questionnaires and reminders to review alcohol consumption feedback (see the Message section for more information). Participants in both arms will complete additional questionnaires on their mood and general mental health when responding to the weekly questionnaires. These responses will be used to personalize the content of the app and push notifications and SMS text messaging delivered to the intervention arm only.

Participants in both arms will be asked to use the app for 28 days. After which, they can continue to use the app; however, they will not receive personalized messaging, and the data collected after this point will not be used in the analyses. However, participants will receive a reminder message on day 84 and day 168 to complete questionnaires.

### Messaging: Push Notifications and SMS Text Messaging

In addition to the *Drinks*:Ration app, participants will receive personalized push notifications and SMS text messaging that provide prompts to use the drinks diary, complete questionnaires, suggest alternative behaviors, provide feedback on goals, and promote a healthy lifestyle. A bank of personalized messages (both for SMS text messages and push notifications) informed by 180 tailored messages developed previously (which are informed by the Health Action Process Approach framework and targeted toward specific BCTs) [49] will be used.

*Drinks*:Ration uses baseline and contiguous measurements (day 7/14/21) to inform the type of message a participant receives to provide an individual participant-centric approach. Baseline measurements are used to identify suitable messages, *and as a participant engages with Drinks*:Ration, continuous measurements, including questionnaires (baseline and weekly questionnaires) and the drinks diary, are used to reflect current behavior and attitude. The messages cover a wide range of topics to target beliefs and motivations, with the primary aim of increasing the participant’s awareness of their drinking habits and behaviors. The messages are divided into 3 categories:

1. Tailored: personalized to drinking habits, baseline, and weekly questionnaires;
2. Tailored and triggered: tailored to baseline and contiguous measurements and a specific event occurring; and
3. Targeted (generic): sent on specific days to highlight inactivity, as a reminder to complete a questionnaire, or to alter participants to a new feature.

A participant can receive a maximum of 20 messages over the 28-day period, with a maximum of 2 messages in a single day. The system automatically decides when a message should be sent and the mode of delivery (push notification or SMS text messaging). A list of targeted (generic) messages to be sent to participants is defined in Table 1; except the messages listed in this table, participants in the control arm will receive no other messages. Participants in both arms will receive a generic message designed to promote retention of the *Drinks*:Ration app to allow for follow-up data collection. This will be sent monthly.

**Table 1:** A list of targeted (generic) messages and the day on which they are sent.

Day	Message content	Study arm
0	Title: Welcome to *Drinks*:Ration Message: Your signup is complete. Remember to log last week’s alcohol consumption	Intervention
0	Title: Welcome to *Drinks*:Ration Message: Your signup is now complete	Control
2	Title: Time to set a goal? Message: Why not set a goal to reduce the amount you drink? You can start now by clicking on the *goals* tab in the app…	Intervention
7	Title: Drinking Advice Message: Remember to open *Drinks*:Ration for advice on how to make your alcohol consumption	Control
13	Title: Remember the diary Message: Completing the drinks diary each day allows you to see how well you are doing!	Intervention
14	Title: Let us know… Message: Remember to open the app to tell us how you’re doing	Intervention and control
18	Title: Remember to monitor Message: Monitoring what you drink is proven to help you cut down—why not start fresh this week?	Intervention
21	Title: Be aware! Message: It is good to be aware of what you drink. Monitoring your alcohol consumption really can help your health!	Intervention and control
23	Title: Think… Message: Using Drinks:Ration may have changed some of the drinks you usually have. Try and think how you can keep it up!	Intervention
28	Title: Let us know… Message: Please let us know how you’re getting on!	Intervention and control
56	Title: Keep checking in… Message: Please remember to open the Drinks:Ration app	Intervention and control
84	Title: Let us know… Message: Please let us know how you’re getting on!	Intervention and control
112/140	Title: Keep checking in… Message: Please remember to open the Drinks:Ration app	Intervention and control
168	Title: Let us know… Message: Please let us know how you’re getting on!	Intervention and control

### Measures

A summary of the measures and data collection timepoints in this study are detailed in Table 2.

#### Baseline Measures

Upon successful registration, participants will complete a baseline questionnaire to assess physical and mental health, health status, resource utilization (eg, visits to hospital because of alcohol, and days in hospital because of alcohol), and sociodemographics (eg, age, gender, ethnicity, employment status, and occupation).

**Table 2:** Summary of measures and data collection timepoints.

Day or measure		0		7		14		21		28		84		168	
**Questionnaires (Multimedia Appendix 2)**															
	Informed consents		I^a^/C^b^		—^c^		—		—		—		—		—
	Sociodemographic		I/C		—		—		—		—		—		—
	Resource utilization		I/C		—		—		—		I/C		I/C		I/C
	Depression (PHQ2^d^) [53]		I/C		I/C		I/C		I/C		I/C		I/C		I/C
	Anxiety (GAD2^e^) [54]		I/C		I/C		I/C		I/C		I/C		I/C		I/C
	International Trauma Questionnaire for PTSD^f^ [55]		I/C		—		—		—		I/C		I/C		I/C
	Readiness to Change Ruler [56]		I/C		—		—		—		I/C		I/C		I/C
	Self-Efficacy Ruler [56]		I/C		—		—		—		I/C		I/C		I/C
	Alcohol Use Disorder Identification Test [57]		I/C		—		—		—		I/C		I/C		I/C
	World Health Organization Quality of Life-BREF [58]		I/C		—		—		—		I/C		I/C		I/C
	7-day Timeline Follow-Back for alcohol consumption [43]		I/C		—		—		—		I/C		I/C		I/C
**Usability evaluation**															
	Qualitative interviews^g^		—		—		—		—		I/C		—		—
	mHealth App Usability Questionnaire [59]		—		—		—		—		I/C		—		—
**Remote data collection**															
	Wearable sensors^h^		I/C		I/C		I/C		I/C		I/C		I/C		I/C
	Smartphone sensors^h^		I/C		I/C		I/C		I/C		I/C		I/C		I/C

^a^I: Intervention arm.

^b^C: Control arm.

^c^—: no data is collected during this period.

^d^PHQ2: Patient Health Questionnaire 2-item.

^e^GAD2: Generalized Anxiety Questionnaire 2-item.

^f^PTSD: posttraumatic stress disorder.

^g^20 participants will be invited to participate in a qualitative interview.

^h^Additional participant consent required.

#### Outcome Measures

The primary outcome measure is the change between self-reported alcohol consumption as measured by the 7-day TLFB [43] over the previous 7 days between baseline (day 0) and 3-month follow-up (day 84). Participants will be asked to report how many drinks they consumed over the last 7 days as well as the type of drink they consumed on each day. Using the standard unit of measurements (Multimedia Appendix 3), weekly alcohol consumption will be determined by summing the number of units assigned to each drink. The TLFB has been extensively used to assess alcohol consumption and has demonstrated good test-retest reliability [43,60]. Secondary outcome measures will be (1) change in the baseline to follow-up AUDIT score and (2) change in the baseline to follow-up World Health Organization Quality of Life-BREF (WHOQOL-BREF) score to assess quality of adjusted life years. Process evaluation measures, following the model by Donabedian for evaluating quality of care [61], will be (1) app use (number of app engagement, screen views, and user retention) and (2) usability ratings as measured by the mHealth App Usability Questionnaire (MAUQ) [59]. A 3-month follow-up period was selected to assess the short-term benefits of the intervention on participants. The primary and secondary outcomes will also be reassessed at 6-month follow-up (day 168) to assess the longer term benefits of the intervention and will be reported as a secondary outcome.

### Study Debrief

At the end of the follow-up period, a random selection of 20 participants (10 from each study arm) will be invited to take part in a 60-min *debrief* session. The aim of this is to understand more about their use and experience of the *Drinks*:Ration app. During the session, participants will be asked to share their views on acceptability and usability and what can be done to improve their experiences. This will be done via telephone and transcript generated and used for analysis. Participants taking part in the debrief session will have the opportunity to view and discuss their own data.

### Study Withdrawal and Consent

**Figure 2:**
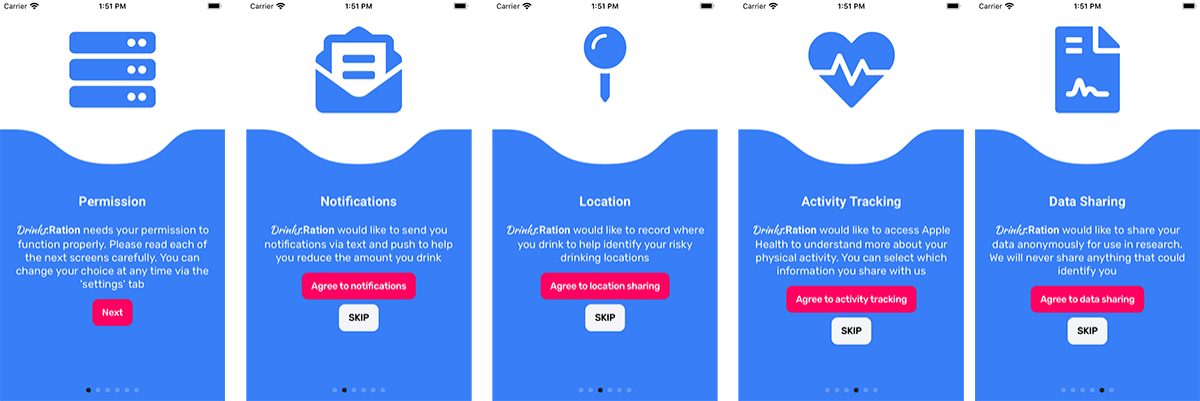
Consent flow screenshot examples presented to all participants of the study.

Informed consent will be sought from all participants via the *Drinks*:Ration app before the collection of any personal data, and they will be informed from the outset that they can withdraw from the study at any time. Individual optional consent (screenshots presented in Figure 2) will be sought for access to the GPS location, sending of push notifications, or SMS text messaging. In addition, to promote open science, consent will be sought from participants to share their data anonymously for research purposes. These data will be released upon completion of the study. Only the questionnaire response, remote measurement technology data, and alcohol consumption statistics will be shared. All data will be deidentified, and no personal data will be released. Participants can change optional consents at any time via the *settings* page of the app until analyses are performed at which point data will be extracted and analyzed.

**Figure 3:**
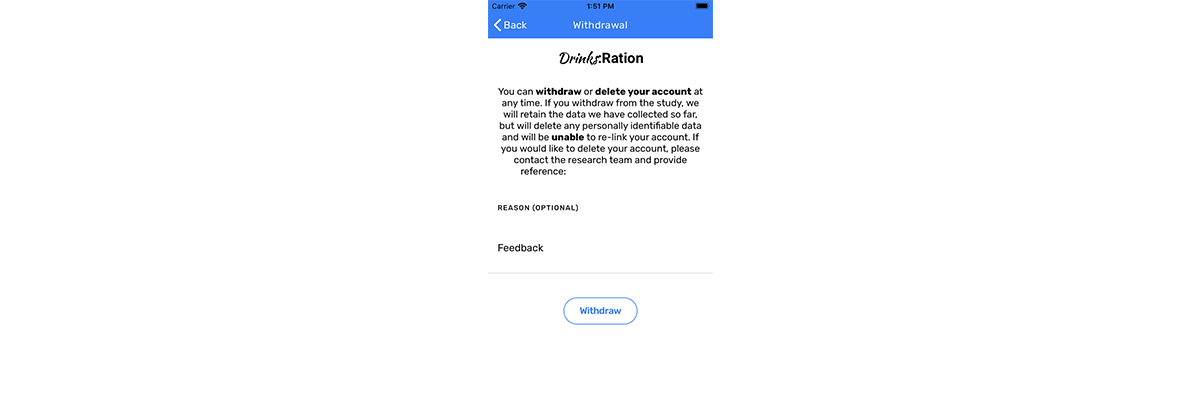
Participant withdrawal screen with includes the ability to delete account or withdraw from the study.

Participants can withdraw from the study at any time within the *Drinks*:Ration app (Figure 3). Participants can choose to withdraw from the study but allow the research team to use the data or delete their account by contacting the research team. We anticipate several reasons for withdrawal from the study, such as:

- Participant choosing to no longer take part in the study: participants will be informed via the *Drinks*:Ration app that participation is voluntary and that they can withdraw from the study at any point without providing a reason.
- Adverse event: participants may be withdrawn by the research team in the event of an adverse event, protocol violation, technical, administrative, or other reason(s).

In the event that participants choose to withdraw from the study, we will provide prompt for a reason as to why they are withdrawing; however, providing a reason will be optional. Participants will be asked to uninstall the *Drinks*:Ration app. All data, including those from withdrawn participants, except the anticipated rare request for their data to be deleted, will be included in the final analysis.

### Adverse Events

It is not expected that participation in this study will lead to an increase in alcohol consumption. Nevertheless, in an adverse event, defined in this study as participants reporting (via the drinks diary or during contact with the research team) that they have consumed more than 25 UK units of alcohol within a 24-hour period, participants will be contacted by the research team, which includes the clinicians (Dr Dominic Murphy, Clinical Psychologist, Head of Research, Combat Stress and Dr Cerisse Gunasinghe, Counseling Psychologist, King’s College London), to perform a clinical interview and risk assessment. If concerns remain, a signposting booklet to relevant charities will be provided to the participants. The study clinicians may register an adverse event and withdraw the participants from the study; however, any data collected before withdrawal will be used for analysis.

### Follow-Up Procedure and Data Monitoring

The research team will monitor incoming data to ensure that the app is functioning correctly. The research team will not contact participants outside automatic messaging of the app unless they reach out to the research team with queries, for example technical issues, or if they are unsure of how to perform a task with the app.

### Additional Data Collection

Additional data will be collected during this study for future research. Participants can opt out for additional data collection at any time.

#### Physical Activity Data Streams

Individual consent will be sought from participants at baseline (day 0) to collect physical activity data using Google Fit and Apple Health Application Programming Interface. The information to be extracted includes heart rate, distance travelled, activities, height, and weight. The aim of this study is to assess the feasibility of monitoring participants who consume alcohol at a hazardous or harmful level. The findings of this study will be reported elsewhere.

### Statistical and Data Analysis Plan

Statistical analysis will be performed after completing the data-collection phase. No interim analysis will be undertaken. The threshold for statistical significance will be *P*=.05, and effect sizes will be reported. Descriptive statistics (eg, demographics and response rate) and independent sample *t* tests and chi-square tests will be carried out to explore and identify potential differences between the intervention and control arms at follow-up. An intention-to-treat method will be used for primary outcome analysis such that those who are lost to follow-up will be retained in the primary analysis. Multiple imputation will be performed to estimate missing data, where appropriate. The primary outcome analysis will examine whether there is a statistically significant difference between the intervention and control arms on change in self-reported TLFB UK units consumed (as stated earlier, a difference of 4 UK units will be considered meaningful). Repeated-measures mixed modeling analyses will be conducted to examine the primary hypothesis that those who receive the full functionality of *Drinks*:Ration will report a greater reduction in alcohol consumption compared with control participants from baseline to 3-month follow-up (day 84). For the secondary outcomes, changes in the AUDIT score and WHOQOL-BREF computed quality of adjusted life years will be assessed using repeated-measures mixed modeling. These analyses will be repeated to assess changes between baseline and 6-month follow-up and will serve as a secondary outcome to assess the longer-term impact of the intervention on participants. These analyses will be reported as secondary outcomes.

For process evaluation measures, app utilization will be assessed using descriptive statistics of frequency of engagement using a previously published procedure [62]. This will include the number of times the app is initialized (started when not running in the background), the average session duration (time spent using the app overall and for each page), the number of times a participant performs an interaction (synchronize data, add a drink, or add a message rating), and the number of weeks in which participants remain engaged with the app. Participant engagement is defined as having at least 3 interactions in a 7-day period, other than receiving push notifications or SMS text messages, and will be used as a proxy for usability. An independent sample analysis will be carried out to explore and identify differences between the intervention and control arms. Usability of *Drinks*:Ration will also be reported using descriptive statistics and thematic analysis of *debrief* sessions. Finally, analyses will be carried out to identify differences between the intervention and control arms in app experience (as measured by MAUQ). In the pursuit of open science, data and code syntax will be published alongside reporting of the trial. As this is a single-blinded RCT, an independent researcher (who will be blinded to allocation) will review all analyses and data before publication. This study will be reported following the Template for Intervention Description and Replication [63] and CONSORT (Consolidated Standards of Reporting Trials) checklist [64].

## Results

Development of *Drinks*:Ration is complete. Owing to the lockdown as a result of COVID-19, the RCT will start in October 2020. As participants can start at different timepoints, we aim to complete all data collection by October 2021. The results of this study will be communicated via publication.

## Discussion

Alcohol misuse is a persistent problem in the UK AF, with estimates forecasting that more than 50% of those who have left the AF meet the criteria for hazardous alcohol use. This is almost double that of the general population [65]. New and innovative modes of delivery and digital interventions are required to meet this demand [66]. At present, there are no smartphone-based alcohol interventions targeted toward the AF to reduce alcohol misuse, with estimates indicating that more than 50% of veterans misuse alcohol use [3]. Therefore, we have developed a theory-driven and user-centered smartphone app, which may help fill this gap in treatment. However, its efficacy, usability, and functionality need to be ascertained in a fully powered RCT. This study protocol describes the design of an RCT to determine the efficacy of *Drinks*:Ration within a treatment-seeking veteran population.

To our knowledge, this is one of the first studies to examine the efficacy of a smartphone app to reduce hazardous/harmful alcohol consumption in a treatment-seeking veteran population that has been developed based on empirical evidence, development guidelines, and co-design. Although this study uses a veteran sample recruited from Combat Stress, it is expected that the findings will be generalizable to the wider veteran community, as Combat Stress is a countrywide organization.

Previous research has shown that veteran alcohol misuse is often highly comorbid with other mental health conditions, which has been shown to have a negative impact on health treatment utilization and that veterans attend fewer health appointments [11]. The use of a smartphone app combined with personalized messaging and formative feedback may provide a cost-effective strategy to deal with the barriers to improving drinking behaviors and increasing mental health care adherence in the UK AF community. These proposed methods may build self-efficacy and promote behavior change in reducing alcohol intake. A novel aspect of this study, which has the potential to improve health care engagement and utilization, is the use of push notifications combined with SMS text messages to support behavior change. To our knowledge, this is one of the first large trials of a text messaging intervention to promote reductions in alcohol consumption in the AF community.

A key strength of this study is the collaboration between the charitable sector, academia, and the UK AF, which has enabled the development of *Drinks*:Ration. A further strength includes remote delivery via smartphones, so that there will be no issues with availability or geographical limitations for participants. The app can also be used on iOS and Android devices. However, our app requires an active data connection for the app to function when logging questionnaires to generate normative feedback. This is to operate the personalization algorithms. We anticipate several challenges in this study. First, our ability to recruit and retain sufficient number of participants to power our analysis, as it is known that many of those who have alcohol-related problems are reluctant to seek help to ameliorate their problems. We planned our recruitment approach following the best practice to mitigate this issue. Further, the app has been designed to promote its active use, with frequent reminders, which is expected to promote adherence to the app. Second, we expect that some participants may encounter technical issues related to the app (eg, unable to log drinks and unable to receive notifications) or the mobile device. To mitigate potential technical or mobile device issues, we have undertaken extensive testing across a range of popular mobile devices and we will allow participants to provide in-app feedback that will be regularly monitored by the research team. Third, the impact of COVID-19 on the study, participant recruitment, engagement, or attrition is unknown. The research team will monitor the situation and handle issues as they arise. Finally, we acknowledge that our sample comprises participants who may be undergoing active treatment. To mitigate this risk, we will undertake statistical analyses to compare the control and intervention arms, and, where identified, these differences will be highlighted when reporting the trial.

## Appendix

Multimedia Appendix 1 Behaviour Change Technique assignment to each *Drinks*:Ration component.

Multimedia Appendix 2 Questionnaire mark-up.

Multimedia Appendix 3 Drinks Menu Alcohol Unit Assignment.

## Abbreviations

AF: armed forces
AUDIT: Alcohol Use Disorders Identification Test
BCT: behavior change technique
MAUQ: mHealth App Usability Questionnaire
NHS: National Health Service
NIHR: National Institute for Health Research
PTSD: posttraumatic stress disorder
QR: quick response
RCT: randomized controlled trial
TLFB: Timeline Follow-back for alcohol consumption
WHOQOL-BREF: World Health Organization Quality of Life-BREF
