# Supplementary material for: Evaluating the Efficacy of a Mobile App (Drinks:Ration) and Personalized Text and Push Messaging to Reduce Alcohol Consumption in a Veteran Population: Protocol for a Randomized Controlled Trial
Source: JMIR Res Protoc. 2020 Oct 2;9(10):e19720. doi: 10.2196/19720 (PMC7568221; doi:10.2196/19720)
Supplement: Multimedia Appendix 1 [file resprot_v9i10e19720_app1.docx]

**Appendix 1: Behaviour Change Technique assignment to each *Drinks*:Ration component**

| **COMPONENT** | **INTERVENTION ARM** | **CONTROL ARM** |
| --- | --- | --- |
| **DRINKS DIARY**  *Allows participants to record alcohol intake and drink free days.* | BCT 2.3: Self-monitoring of behaviour | - |
| **ALCOHOL CONSUMPTION USING TIMELINE FOLLOWBACK**  *Allows the participant to retrospectively record their alcohol consumption in the week prior to beginning to use the app.* | BCT 2.3 Self-monitoring of behaviour | BCT 2.3: Self-monitoring of behaviour |
| **GENERIC MESSAGES**  *Generic messages sent to both arms, e.g. to highlight features and questionnaires which are due.* | BCT 7.1: Prompts/cues  BCT 5.1: Information about health consequences | BCT: 7.1 Prompts/cues  BCT 5.1: Information about health consequences |
| **PERSONALISED MESSAGES**  *Tailored messages are sent to participants to prompt use of app functions, e.g. drinks diary.* | BCT 3.2: Social support (practical)  BCT 7.1: Prompts/cues  BCT 8.2: Behaviour substitution  BCT 10.4: Social reward | - |
| **PERSONALISED**  **FEEDBACK**  *Tailored feedback is provided including comparing alcohol use to the general population, Armed Forces community and other users of Drinks:Ration.* | BCT 2.2: Feedback on behaviour  BCT 5.2: Salience of consequences  BCT 6.2: Social comparison | - |
| **GOVERNMENT GUIDANCE FEEDBACK**  *UK guidance on alcohol consumption and low risk drinking.* | BCT 5.1: Information about health consequences  BCT 9.1: Credible source | BCT 5.1: Information about health consequences  BCT 9.1: Credible source |
| **DRINKING ZONES**  *Shows on a map all locations where drinks have been added to drinks diary.* | BCT 12.3: Avoidance/reducing exposure to cues for the behaviour | - |
| **GOAL SETTING**  *Participants can set goals, they receive visual feedback on progress towards achieving these goals.* | BCT 1.1: Goal setting (behaviour)  BCT 1.2: Problem solving  BCT 1.3: Goal setting (outcome)  BCT 1.4: Action planning  BCT 1.5: Review behaviour goals  BCT 2.4: Self-monitoring of outcome(s) of behaviour  BCT 8.2: Behavioural substitution  BCT 8.4: Habit reversal  BCT 15.3: Focus on past success | - |
| **DRINKING ADVICE**  *Advice/tips to help the participant reduce their consumption, e.g. “take a break. plan some drink free days”.* | BCT 8.2: Behaviour substitution  BCT 8.4: Habit reversal  BCT 12.2: Restructuring the social environment | - |
| **TIMELINE**  *Graphs to display alcohol units, price, calories and total drinks over 1, 2 and 4 weeks.* | BCT 2.2: Feedback on behaviour  BCT 7.1: Prompts/cues | - |
| **DRINKS IN PIXELS**  *Provides a visual representation of drink free days versus drinking days over one year.* | BCT 2.2: Feedback on behaviour  BCT 15.3: Focus on past success | - |
