# Supplementary material for: Evaluating the Efficacy of a Mobile App (Drinks:Ration) and Personalized Text and Push Messaging to Reduce Alcohol Consumption in a Veteran Population: Protocol for a Randomized Controlled Trial
Source: JMIR Res Protoc. 2020 Oct 2;9(10):e19720. doi: 10.2196/19720 (PMC7568221; doi:10.2196/19720)
Supplement: Multimedia Appendix 2 [file resprot_v9i10e19720_app2.docx]

**Appendix 2. Questionnaire Mark-up**

Eligibility Questionnaire

1. Are you currently serving in the UK Armed Forces?
   1. 1. No, I have left service
   2. 2. Yes, I am a reservist
   3. 3. Yes
2. 7-day Time-Line Follow Back for Alcohol Consumption

Baseline Socio-demographics Questionnaire

1. What is your current gender?
   1. 1. Male
   2. 2. Female
   3. 3. Other
2. What is your ethnic group?
   1. 1. White
   2. 2. Mixed/multiple ethnic groups
   3. 3. Asian/Asian British/British Chinese
   4. 4. Black African/Caribbean/Black British
   5. 5. Other ethnic Group
3. How old are you?
4. What is your current occupation?
5. How long did you serve in the Armed Forces?
6. Which best describes your reason for leaving
   1. 1. Completed term of service
   2. 2. Better employment prospects in civilian life
   3. 3. Impact of service life on family
   4. 4. Work not exciting or challenging
   5. 5. Dissatisfaction with pay
   6. 6. Lack of promotion prospects
   7. 7. Difficult to plan life outside of work
   8. 8. Due to deployment
   9. 9. Pressure on family
   10. 10. Didn’t want to be aware from home
   11. 11. My service was terminated
   12. 12. Health problems
   13. 13. Pregnancy
   14. 14. Accomplished everything I wanted
7. Are you a reservist?
   1. 1. Yes
   2. 0. No
8. What is your current email? This will be used to reminder you of study questionnaires.
   1. [FREE TEXT]
9. Would you be interested in being contacted in future regarding follow up studies?
   1. 1. Yes
   2. 0. No

Resource Utilisation

In the past 28 days have you had:

1. Any days off work due to alcohol?
2. Any workdays affected by alcohol?
3. Any accidents due to alcohol (please provide brief description)?
4. Any visits to A&E due to alcohol?
5. Any days in hospital due to alcohol?
6. Any police contacts due to alcohol?
7. Any physical fights due to alcohol?
8. Any GP visits due to alcohol?

Depression (PHQ2)

Over the last week, how often have you been bothered by the following problems?

| 1. Little interest or pleasure in doing things | Not at all (0) | Several days (1) | More than half the days (2) | Nearly every day (3) |
| --- | --- | --- | --- | --- |
| 2. Feeling down, depressed or hopeless | Not at all (0) | Several days (1) | More than half the days (2) | Nearly every day (3) |

Anxiety (GAD2)

Over the last week1, how often have you been bothered by the following problems?

| 1. Feeling nervous, anxious or on edge | Not at all (0) | Several days (1) | More than half the days (2) | Nearly every day (3) |
| --- | --- | --- | --- | --- |
| 2. Not being able to stop or control worrying | Not at all (0) | Several days (1) | More than half the days (2) | Nearly every day (3) |

International Trauma Questionnaire for PTSD

Have you been bothered by these problems in the past month?

| 1. Having upsetting dreams that replay part of the  experience or are clearly related to the experience? | Not at all (0) | A little bit (1) | Moderately (2) | Quite a bit (3) | Extremely (4) |
| --- | --- | --- | --- | --- | --- |
| 2. Having powerful images or memories that  sometimes come into your mind in which you feel the experience is happening again in the here and now? | Not at all (0) | A little bit (1) | Moderately (2) | Quite a bit (3) | Extremely (4) |
| 3. Avoiding internal reminders of the experience (for example, thoughts, feelings, or physical sensations)? | Not at all (0) | A little bit (1) | Moderately (2) | Quite a bit (3) | Extremely (4) |
| 4. Avoiding external reminders of the experience (for example, people, places, conversations, objects,  activities, or situations)? | Not at all (0) | A little bit (1) | Moderately (2) | Quite a bit (3) | Extremely (4) |
| 5. Being “super-alert”, watchful, or on guard? | Not at all (0) | A little bit (1) | Moderately (2) | Quite a bit (3) | Extremely (4) |
| 6. Feeling jumpy or easily startled? | Not at all (0) | A little bit (1) | Moderately (2) | Quite a bit (3) | Extremely (4) |
| In the past month have the above problems | | | | | |
| 7. Affected your relationships or social life? | Not at all (0) | A little bit (1) | Moderately (2) | Quite a bit (3) | Extremely (4) |
| 8. Affected your work or ability to work? | Not at all (0) | A little bit (1) | Moderately (2) | Quite a bit (3) | Extremely (4) |
| 9. Affected any other important part of your life such as parenting, or school or college work, or other important activities? | Not at all (0) | A little bit (1) | Moderately (2) | Quite a bit (3) | Extremely (4) |

Readiness to Change Ruler

Using the ruler below, indicate how ready you are to make a change to your drinking. If you are not at all ready, you would select 0 and if you are already trying hard to make the change, you would select 10.

Self-efficacy Ruler

Using the ruler shown below, indicate how confident you are about making a change to your drinking. If you are not at all confident about making the change, you would select 0. If you are very confident about making the change, you would select 10.

Alcohol Use Disorder Identification Test

| 1. How often do you have a drink containing alcohol? | (0) Never | (1) Monthly or less | (2) 2 to 4 times a month | (3) 2 to 3 times a week | (4) 4 or more times a week |
| --- | --- | --- | --- | --- | --- |
| 2. How many drinks containing alcohol do you have on a typical day when you are drinking? | (0) 1 or 2 | (1) 3 or 4 | (2) 5 or 6 | (3) 7, 8, or 9 | (4) 10 or more |
| 3. How often do you have six or more drinks on one  occasion? | (0) Never | (1) Less than monthly | (2) Monthly | (3) Weekly | (4) Daily or almost daily |
| 4. How often during the last year have you found  that you were not able to stop drinking once you  had started? | (0) Never | (1) Less than monthly | (2) Monthly | (3) Weekly | (4) Daily or almost daily |
| 5. How often during the last year have you failed to do what was normally expected from you  because of drinking? | (0) Never | (1) Less than monthly | (2) Monthly | (3) Weekly | (4) Daily or almost daily |
| 6. How often during the last year have you needed  a first drink in the morning to get yourself going after a heavy drinking session? | (0) Never | (1) Less than monthly | (2) Monthly | (3) Weekly | (4) Daily or almost daily |
| 7. How often during the last year have you had a  feeling of guilt or remorse after drinking? | (0) Never | (1) Less than monthly | (2) Monthly | (3) Weekly | (4) Daily or almost daily |
| 8. How often during the last year have you been  unable to remember what happened the night  before because you had been drinking? | (0) Never | (1) Less than monthly | (2) Monthly | (3) Weekly | (4) Daily or almost daily |
| 9. Have you or someone else been injured as a  result of your drinking? | (0) No | (2) Yes, but not in the last year | (4) Yes, during the last year |  |  |
| 10. Has a relative or friend or a doctor or another  health worker been concerned about your drinking or suggested you cut down? | (0) No | (2) Yes, but not in the last year | (4) Yes, during the last year |  |  |

World Health Organisation Quality of Life-BREF

Please read each question, assess your feelings, and select the most suitable answer to you currently.

| 1. How would you rate your quality of life? | (1) Very poor | (2) Poor | (3) Neither poor nor good | (4) Good | (5) Very good |
| --- | --- | --- | --- | --- | --- |
| 2. How satisfied are you with your health? | (1) Very dissatisfied | (2) Dissatisfied | (3) Neither satisfied nor dissatisfied | (4) Satisfied | (5) Very satisfied |
| 3. To what extent do you feel that physical pain prevents you from doing what you need to do? | (5) Not at all | (4) A little | (3) A moderate amount | (2) Very much | (1) An extreme amount |
| 4. How much do you need any medical  treatment to function in your daily life? | (5) Not at all | (4) A little | (3) A moderate amount | (2) Very much | (1) An extreme amount |
| 5. How much do you enjoy life? | (1) Not at all | (2) A little | (3) A moderate amount | (4) Very much | (5) An extreme amount |
| 6. To what extent do you feel your life to  be meaningful? | (1) Not at all | (2) A little | (3) A moderate amount | (4) Very much | (5) An extreme amount |
| 7. How well are you able to concentrate? | (1) Not at all | (2) A little | (3) A moderate amount | (4) Very much | (5) Extremely |
| 8. How safe do you feel in your daily life? | (1) Not at all | (2) A little | (3) A moderate amount | (4) Very much | (5) Extremely |
| 9. How healthy is your physical environment? | (1) Not at all | (2) A little | (3) A moderate amount | (4) Very much | (5) Extremely |
| 10. Do you have enough energy for everyday life? | (1) Not at all | (2) A little | (3) Moderately | (4) Mostly | (5) Completely |
| 11. Are you able to accept your bodily appearance? | (1) Not at all | (2) A little | (3) Moderately | (4) Mostly | (5) Completely |
| 12. Have you enough money to meet your needs? | (1) Not at all | (2) A little | (3) Moderately | (4) Mostly | (5) Completely |
| 13. How available to you is the information that you need in your day-to-day life? | (1) Not at all | (2) A little | (3) Moderately | (4) Mostly | (5) Completely |
| 14. To what extent do you have the opportunity for leisure activities? | (1) Not at all | (2) A little | (3) Moderately | (4) Mostly | (5) Completely |
| 15. How well are you able to get around? | (1) Very poor | (2) Poor | (3) Neither poor nor good | (4) Good | (5) Very good |
| 16. How satisfied are you with your sleep? | (1) Very dissatisfied | (2) Dissatisfied | (3) Neither satisfied nor dissatisfied | (4) Satisfied | (5) Very satisfied |
| 17. How satisfied are you with your ability to perform your daily living activities? | (1) Very dissatisfied | (2) Dissatisfied | (3) Neither satisfied nor dissatisfied | (4) Satisfied | (5) Very satisfied |
| 18. How satisfied are you with your capacity  for work? | (1) Very dissatisfied | (2) Dissatisfied | (3) Neither satisfied nor dissatisfied | (4) Satisfied | (5) Very satisfied |
| 19. How satisfied are you with yourself? | (1) Very dissatisfied | (2) Dissatisfied | (3) Neither satisfied nor dissatisfied | (4) Satisfied | (5) Very satisfied |
| 20. How satisfied are you with your personal relationships? | (1) Very dissatisfied | (2) Dissatisfied | (3) Neither satisfied nor dissatisfied | (4) Satisfied | (5) Very satisfied |
| 21. How satisfied are you with your sex life? | (1) Very dissatisfied | (2) Dissatisfied | (3) Neither satisfied nor dissatisfied | (4) Satisfied | (5) Very satisfied |
| 22. How satisfied are you with the support you get from your friends? | (1) Very dissatisfied | (2) Dissatisfied | (3) Neither satisfied nor dissatisfied | (4) Satisfied | (5) Very satisfied |
| 23. How satisfied are you with the conditions of your living place? | (1) Very dissatisfied | (2) Dissatisfied | (3) Neither satisfied nor dissatisfied | (4) Satisfied | (5) Very satisfied |
| 24. How satisfied are you with your access to health services? | (1) Very dissatisfied | (2) Dissatisfied | (3) Neither satisfied nor dissatisfied | (4) Satisfied | (5) Very satisfied |
| 25. How satisfied are you with your transport? | (1) Very dissatisfied | (2) Dissatisfied | (3) Neither satisfied nor dissatisfied | (4) Satisfied | (5) Very satisfied |
| 26. How often do you have negative feelings such as blue mood, despair, anxiety, depression? | (5) Never | (4) Seldom | (3) Quite often | (2) Very often | (1) Always |

mHealth App Usability Questionnaire

| 1. The app was easy to use. | (0) N/A | (1) Disagree) | (2) | (3) | (4) Neutral | (5) | (6) | (7) Agree |
| --- | --- | --- | --- | --- | --- | --- | --- | --- |
| 2. It was easy for me to learn to use the app | (0) N/A | (1) Disagree) | (2) | (3) | (4)  Neutral | (5) | (6) | (7) Agree |
| 3. I like the interface of the app. | (0) N/A | (1) Disagree) | (2) | (3) | (4)  Neutral | (5) | (6) | (7) Agree |
| 4. The information in the app was well organized,  so I could easily find the information I needed | (0) N/A | (1) Disagree) | (2) | (3) | (4)  Neutral | (5) | (6) | (7) Agree |
| 5. I feel comfortable using this app in social  settings. | (0) N/A | (1) Disagree) | (2) | (3) | (4)  Neutral | (5) | (6) | (7) Agree |
| 6. The amount of time involved in using this app  has been fitting for me. | (0) N/A | (1) Disagree) | (2) | (3) | (4)  Neutral | (5) | (6) | (7) Agree |
| 7. I would use this app again. | (0) N/A | (1) Disagree) | (2) | (3) | (4)  Neutral | (5) | (6) | (7) Agree |
| 8. Overall, I am satisfied with this app. | (0) N/A | (1) Disagree) | (2) | (3) | (4)  Neutral | (5) | (6) | (7) Agree |
| 9. Whenever I made a mistake using the app, I  could recover easily and quickly. | (0) N/A | (1) Disagree) | (2) | (3) | (4)  Neutral | (5) | (6) | (7) Agree |
| 10. This mHealth app provides an acceptable way to  receive healthcare services. | (0) N/A | (1) Disagree | (2) | (3) | (4)  Neutral | (5) | (6) | (7) Agree |
| 11. The app adequately acknowledged and provided information to let me know the progress of my action. | (0) N/A | (1) Disagree | (2) | (3) | (4)  Neutral | (5) | (6) | (7) Agree |
| 12. The navigation was consistent when moving  between screens. | (0) N/A | (1) Disagree | (2) | (3) | (4)  Neutral | (5) | (6) | (7) Agree |
| 13. The interface of the app allowed me to use all  the functions (such as entering information,  responding to reminders, viewing information)  offered by the app. | (0) N/A | (1) Disagree | (2) | (3) | (4)  Neutral | (5) | (6) | (7) Agree |
| 14. This app has all the functions and capabilities I  expected it to have. | (0) N/A | (1) Disagree | (2) | (3) | (4)  Neutral | (5) | (6) | (7) Agree |
| 15. The app would be useful for my health and wellbeing. | (0) N/A | (1) Disagree | (2) | (3) | (4)  Neutral | (5) | (6) | (7) Agree |
| 16. The app helped me manage my health effectively. | (0) N/A | (1) Disagree | (2) | (3) | (4)  Neutral | (5) | (6) | (7) Agree |
