# Supplementary material for: Evaluating the Efficacy of a Mobile App (Drinks:Ration) and Personalized Text and Push Messaging to Reduce Alcohol Consumption in a Veteran Population: Protocol for a Randomized Controlled Trial
Source: JMIR Res Protoc. 2020 Oct 2;9(10):e19720. doi: 10.2196/19720 (PMC7568221; doi:10.2196/19720)
Supplement: Multimedia Appendix 3 [file resprot_v9i10e19720_app3.docx]

**Appendix 3: Drinks Menu Alcohol Unit Assignment**

Based on calculating alcohol unit guidance from the NHS Live Well Service.

| **Type of Drink** | **Number of Unit** |
| --- | --- |
| Single small shot of spirits* (25ml, ABV 40%) | 1 |
| Alcopop (275ml, ABV 5.5%) | 1.5 |
| Small glass of red/white/rosé wine (125ml, ABV 12%) | 1.5 |
| Bottle of lager/beer/cider (330ml, ABV 5%) | 1.7 |
| Can of lager/beer/cider (440ml, ABV 5.5%) | 2 |
| Pint of lower-strength lager/beer/cider (568ml, ABV 3.6%) | 2 |
| Standard glass of red/white/rosé wine (175ml, ABV 12%) | 2.1 |
| Pint of higher-strength lager/beer/cider (568ml, ABV 5.2%) | 3 |
| Large glass of red/white/rosé wine (250ml, ABV 12%) | 3 |

*Gin, rum, vodka, whisky, tequila, sambuca. Large (35ml) single measures of spirits are 1.4 units.
